# Apoptosis signal-regulating kinase 1 mediates striatal degeneration via the regulation of C1q

**DOI:** 10.1038/srep18840

**Published:** 2016-01-05

**Authors:** Kyoung Joo Cho, So Young Cheon, Gyung Whan Kim

**Affiliations:** 1 Department of Neurology, Severance Hospital, Yonsei University College of Medicine, 50 Yonsei-ro, Seoul, South Korea; 2 Department of Anesthesiology and Pain, Severance Hospital, Yonsei University College of Medicine, 50 Yonsei-ro, Seoul, South Korea

## Abstract

Apoptosis signal-regulating kinase-1 (ASK1), an early signaling element in the cell death pathway, has been hypothesized to participate in the pathology of neurodegenerative diseases. The systemic administration of 3-nitropropionic acid (3-NP) facilitates the development of selective striatal lesions. However, it remains unclear whether specific neurons are selectively targeted in 3-NP-infused striatal degeneration. Recently, it has been proposed that complement-mediated synapse elimination may be reactivated aberrantly in the pathology of neurodegenerative diseases. We hypothesized that ASK1 is involved in striatal astrocyte reactivation; reactive astrocyte secretes molecules detrimental to neuron; and striatal neurons are more susceptible to these factors. Our results indicate that striatal astrocyte is reactivated and ASK1 level increases after 3-NP general and chronic infusion. Reactive striatal astrocyte increases TGF-beta differentially to cortex and striatum. ASK1 may be involved in regulation of astrocyte TGF-beta and it is linked to the C1q level in spatial and temporal, and moreover in the earlier stage of progressing striatal neuronal loss. Conclusively the present study suggests that ASK1 mediates 3-NP toxicity and regulates C1q level through the astrocyte TGF-beta. And also it may suggest that C1q level may be a surrogate of prediction marker representing neurodegenerative disease progress before developing behavioral impairment.

The systemic administration of 3-nitropropionic acid (3-NP) facilitates the development of selective striatal lesions. However, it remains unclear whether specific neurons are selectively targeted in 3-NP-infused striatal degeneration. It is well known that 3-NP inhibits succinate dehydrogenase (SDH) and mitochondrial complex II to the same extent in the cerebrum and striatum^1^,^2^, which suggest that 3-NP neurotoxicity is similar in the cerebrum and striatum. 3-NP-induced neurotoxicity has been shown to have the following effects: the exhaustion of adenosine triphosphate, mitochondrial membrane depolarization, dysregulation of intracellular calcium homeostasis, calpain activation, and the release of pro-apoptotic proteins from the mitochondria^3^,^4^,^5^. The neurotoxic mechanism and the reason for the selective vulnerability of the striatum are not yet well understood. Therefore, the molecular mechanisms underlying 3-NP-induced striatal lesions remain to be fully defined^6^. As a crucial component of neuronal loss in neurodegenerative disease, innate immune cascade is emerging^7^. C1q is a component of the complement initiator C1 complex. Excess C1q may lead to an increase in functionally active levels of C1, which results in an increase in the activation of the classical complement pathway^8^. During development, C1q is expressed in the synaptic regions of the developing postnatal central nervous system (CNS)^9^. Based on the brain developmental stage, the complement pathway can trigger either appropriate neuronal development or chronic inflammation, the latter of which is involved in neurodegeneration^9^. The overexpression of C1q in the CNS has been observed in several injury models^10^. In a stroke model, an increase in C1q drives two independent pathophysiological mechanisms involved in the processes of lesion formation and blood-brain barrier (BBB) disruption^11^. C1q plays a role as a pattern recognition receptor^12^ that mediates the clearance of cells undergoing cell death by apoptosis^13^ and can enhance the uptake of apoptotic cells^14^. Exogenous plasma C1q, therefore, may play a role in the initiation of cell activation in microglial cells during the course of CNS diseases that involve BBB impairment and may contribute in an autocrine or paracrine way to maintain and balance microglial activation in the diseased CNS tissue^15^. Neuronal C1q expression is downregulated in the CNS in adulthood^16^, but knock-out mice lacking C1q have sustained defects in CNS synapse elimination^17^.

Apoptosis signal-regulating kinase-1 (ASK1), an early signaling element in the cell death pathway^18^, is activated by reactive oxygen species (ROS)^19^ and is required for ROS-induced apoptosis^20^. Previous studies have indicated that increased levels of activated ASK1 impact the presentation of neurodegenerative diseases^21^. The rationale for targeting ASK1 was based on findings that oxidative stress is causally related to apoptotic neuronal cell death in neurodegenerative lesions associated with abnormal protein fragments and aggregates^22^,^23^. Although many investigations have shown that ASK1 is essentially involved in neuronal cell death triggered by various external injuries and expanded polyglutamine, the relationship between ASK1 and selective striatal degeneration involving innate immunity by the chronic and systemic infusion of 3-NP in mice remains to be elucidated.

Based on the reports mentioned above^9^,^13^,^17^, we assumed as following: when the brain is mildly and chronically exposed to mitochondrial toxins, presynaptic neurons degrade first, and the postsynaptic striatal medium spiny neurons (MSNs) subsequently wither. Accordingly the current study investigated whether increased levels of ASK1 triggered by the mild and chronic infusion of 3-NP modulate neuronal C1q secretion and synapse elimination in the cortex and striatum. In addition, the current study investigated the involvement of astrocyte-derived TGF-beta as an inducing factor for C1q in neurodegenerative striatal cell death.

## Results

### Selective striatal lesions are formed by the systemic infusion of 3-NP in mice

**Figure 1:**
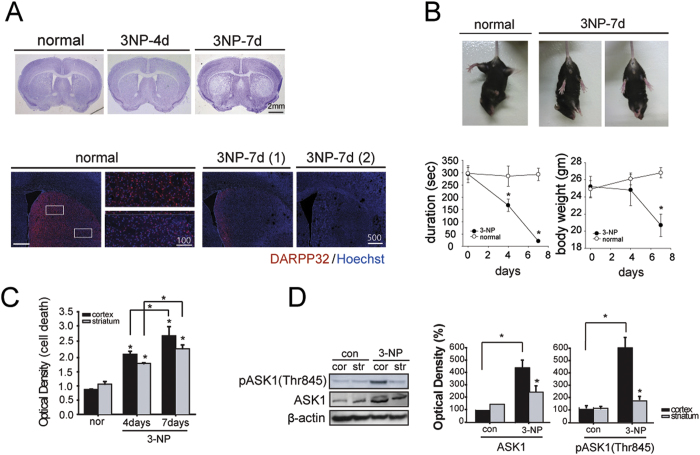
The chronic infusion of 3-NP induces cell death and selective striatal lesions. (**A**) Histological analysis by cresyl violet staining demonstrates selective striatal cell loss at day seven (upper). Striatal neurons stained by DARPP32 are almost undetectable by day seven (lower). (**B**) Mice exhibited hindlimb impairment on day seven (upper). The body weight of 3-NP infused mice decreased gradually (lower, left graph), and mice had trouble staying on the rotarod (lower, right graph). (**C**) Cell death assay present the quantitative values in each region of cortex and striatum. (**D**) Western blot analyses of ASK1 and pASK1 (Thr845, active ASK1) indicate the activation of ASK1 in the cortex following 3-NP chronic infusion. The protein level of ASK1 and pASK1 are shown in the quantified graphs. *p < 0.05; Scale bars: 2 mm, 500 μm, and 100 μm.

The histological evaluation of 3-NP-infused mice (n = 11 each) revealed scant striatal lesions at day four but significant selective striatal cell loss at day seven (Fig. 1A). Immunostaining with dopamine- and cyclic adenosine monophosphate-regulated phosphoprotein (DARPP32), an established striatal neuron marker, revealed that striatal neurons stained with DARPP32 disappeared by day seven (Fig. 1A). Most of the mice exhibited the obvious symptoms of selective striatal loss, such as weight loss and severe motor dysfunction, seven days after systemic 3-NP infusion. The motor function was tested whether the mice were able to adjust to imbalanced postural challenges. Mice infused with 3-NP did not display any neurological deficits until day four. Five days later, however, hindlimb clasping was observed (Fig. 1B) and an adjustment to postural imbalances was failed. On rotarod test, the 3-NP-infusion mice could not remain on the rotarod and body weight was reduced to 70% of the initial weight (Fig. 1B, n = 12 each). The DNA damage in the brain was determined by TUNEL staining at day seven (Fig. 1C, n = 6 each). Apoptotic cell death assay present the quantitative values in each region of cortex and striatum, and it increased over the days following 3-NP infusion (Fig. 1C). To evaluate primary response to damage by 3-NP, both ASK1 and the pASK1 (Thr845, active ASK1), which are found in the MAP kinase pathway, were analyzed by Western blot (Fig. 1D, n = 6 each). The amounts of ASK1 and pASK1were greater in 3-NP-infused mice than normal, and was also present in higher concentrations in the cortex than the striatum in 3-NP-infused mice. The quantified graphs represent normalizing values with ASKl protein amounts.

### The infusion of 3-NP leads to selective striatal neuronal loss and also astrocyte reactivation

**Figure 2:**
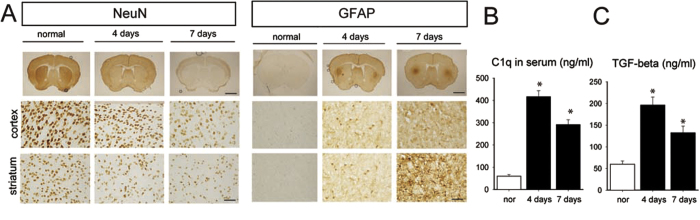
The infusion of 3-NP leads to selective striatal neuronal loss as well as astrocyte reactivation. (**A**) Neuronal loss was detected by NeuN Immunostaining (left). Astrocytes were strongly reactivated in the lateral striatum as observed by GFAP Immunostaining (right). Reactive astrocytes started to increase from day four when the lesion was not yet detected and remained reactivated at day seven when the lesion was definite. Each of the two types of CNS cells responded differently to the 3-NP infusion. (**B**) The ELISA results from 3-NP-infused mouse blood serum revealed that the secretion levels of C1q were increased on day four when the lesion was not yet detectable and on day seven when the lesion was detectable in 3-NP infused mice. (**C**) The results of ELISA for TGF-beta show the similar pattern to that of C1q in 3-NP infused mice. Scale bars: 2 mm and 100 μm.

Neurons stained with NeuN were distributed homogeneously in the cortex and striatum of control animals, whereas sparse neuronal staining was observed in the striatum of 3-NP-infused mice (Fig. 2A, left). Moreover, the neuronal loss was more severe in the striatum than in the cortex seven days after 3-NP infusion. GFAP immunostaining revealed that astrocytes were weakly detected along the cortex and striatum of normal but strongly reactivated in the lateral striatum of 3-NP-infused mice at the boundary between the striatum and cortex from the four days after 3-NP infusion (Fig. 2A, right). Our preliminary data in another study indicated that C1q, a complement factor in the innate immune system, is elevated by brain injury relating to ASK1 expression level (data not shown). ELISA revealed that C1q peaked four days after 3-NP infusion before the lesions were detectable and were reduced by day seven when the lesions were detectable (Fig. 2B). As a candidate for direct factors involving in the regulation of C1q induction, striatal-specific astrocyte reactivation gave hints that an astrocyte-related secreting molecule may be involved in selective striatal lesion formation followed by neuronal factors that impose neuronal loss or degeneration. We assayed TGF-β1 secretion level that is the potential factor of astrocyte-derived molecules in neuronal loss (Fig. 2C). The secretion levels of TGF-β1 demonstrated a similar temporal pattern to those of C1q, with a higher level on four days after 3-NP infusion than on day seven. It presented the same pattern with the C1q secreting pattern and it was preceded to definite lesion formation (Fig. 2B).

### The downregulation of ASK1 prevented 3-NP-induced cell death and subsequently improved neurologic impairment

**Figure 3:**
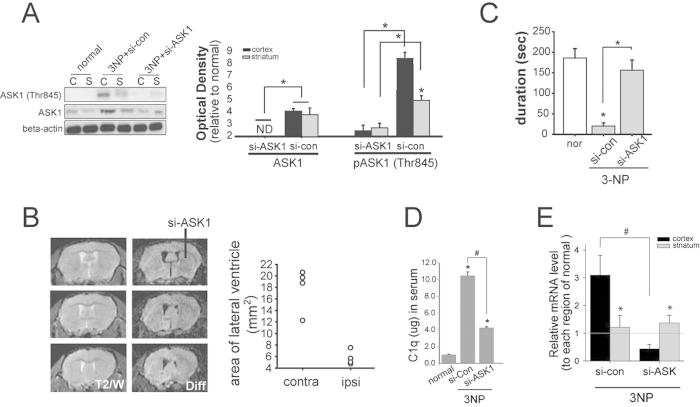
The downregulation of ASK1 prevents 3-NP-induced striatal cell loss and alleviates motor function. (**A**) pASK1 (Thr845) levels decreased as levels of pAkt (Ser473) increased, which indicates that the ASK1 gene was silenced. The semi-quantitative graph shows these results. (**B**) Representative MRIs display diffusion images across seven days of 3-NP-infused mice. ASK1 downregulation was achieved by infusing siRNA-ASK1 into one side of the striatum. Each MR image presents the three different positions from the bregma. A quantitative analysis was conducted to evaluate the area of the lateral ventricle, and is shown in the graph. (**C**) ASK1 gene-silenced 3-NP-infused mice demonstrated improved motor function on the rotarod test. (**D**) The result of ELISA presents C1q secretion level to be reduced by ASK1 silencing in the mouse whole serum infused 3-NP for four days. (**E**) Real time PCR revealed that the C1q transcripts increased in the cortex at day four after 3-NP infusion and significantly decreased by si-ASK1 treatment. Scale bars: 100 μm and 20 μm each. si-ASK1, siRNA for ASK1; si-control, siRNA for non-functional mixed gene; or, normal; cor, cortex; str, striatum; NS, not significant. *p < 0.05.

**Figure 4:**
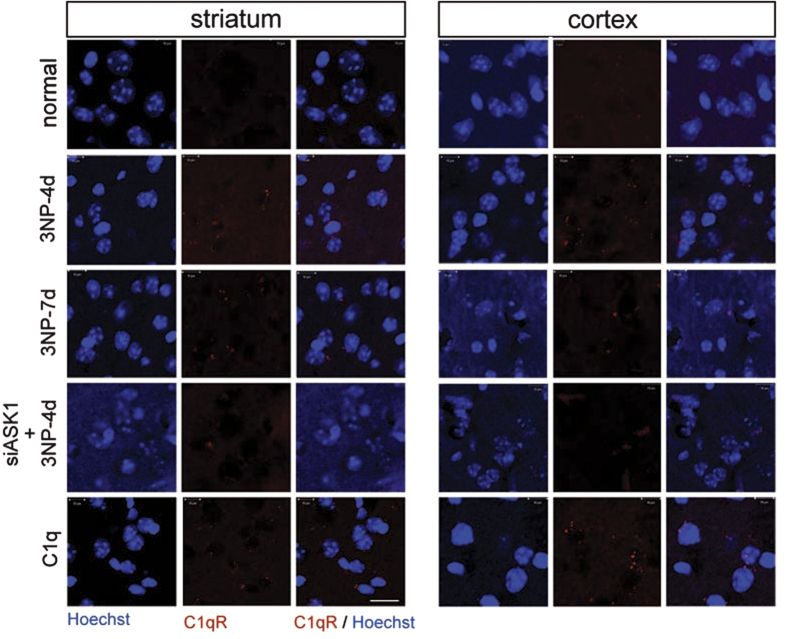
The downregulation of ASK1 restrains C1qR expression. C1qR immunohistochemistry was conducted in the cortex and striatum of 3-NP-infused mice. C1qR-positive cells were detected (red dots) four and seven days after 3-NP infusion. C1qR immunohistochemistry also was performed in ASK1-downregulated and C1q-treated 3-NP-infused mice. Scale bar: 20 μm.

The ASK1 gene was silenced using siRNA for seven days during 3-NP infusion. Primarily the silencing of the ASK1 gene via siRNA administration was sufficient to downregulate ASK1 protein levels, which resulted in the inhibition of pASK1 (Thr845) expression (Fig. 3A, n = 7 each). *In vivo* MRIs of siRNA-ASK1-treated 3-NP-infused mice were performed seven days after the infusion of 3-NP and siRNA (Fig. 3B, n = 4 each). Progressive enlargement was evident in the lateral ventricles on the contralateral side, and striatal atrophy was detected. In contrast, on the ipsilateral side where siRNA-ASK1 was infused, ventricular enlargement and striatal atrophy did not indicate severe regression (Fig. 3B). Apoptotic cell death assay showed that a lower incidence of cell death was detected in the siRNA-ASK1-treated mice than in the siRNA-control-treated mice (data not shown). Consequently, 3-NP-infused mice that received siRNA-ASK1 treatment were able to remain longer on the rotarod than 3-NP-infused siRNA-control-treated mice (Fig. 3C, n = 7 each). The downregulation of ASK1 hampered C1q secretion in the whole blood serum (Fig. 3D, n = 5 each). With cortical and striatal tissue lysates from si-RNA-con and siRNA-ASK1 treated mice, C1q secreting level was also assayed via ELISA. The total level reduced in ASK1 downregulated mice, and it indicated considerable reduction of the C1q level in cortical tissue (Fig. 3E, n = 5 each). To confirm special distribution of C1q, C1qR immunohistochemistry was performed in control, 3-NP-infused, ASK1- downregulated. And it was confirm whether increased C1q leads C1qR and neuronal loss, C1q was treated into striatum of mice. Several C1qR-positive cells were detected in the brains of C1q-treated, and also in four-day-3-NP-infusion mice, which was consistent with the findings of elevated C1q secretion (Fig. 4). Few C1qR-positive cells were detected in the brains of ASK1-downregulated 3-NP-infused mice.

### Astrocyte-secreted TGF-β1 triggers neuronal C1q and degeneration of neuronal dendrites mediated by ASK1

**Figure 5:**
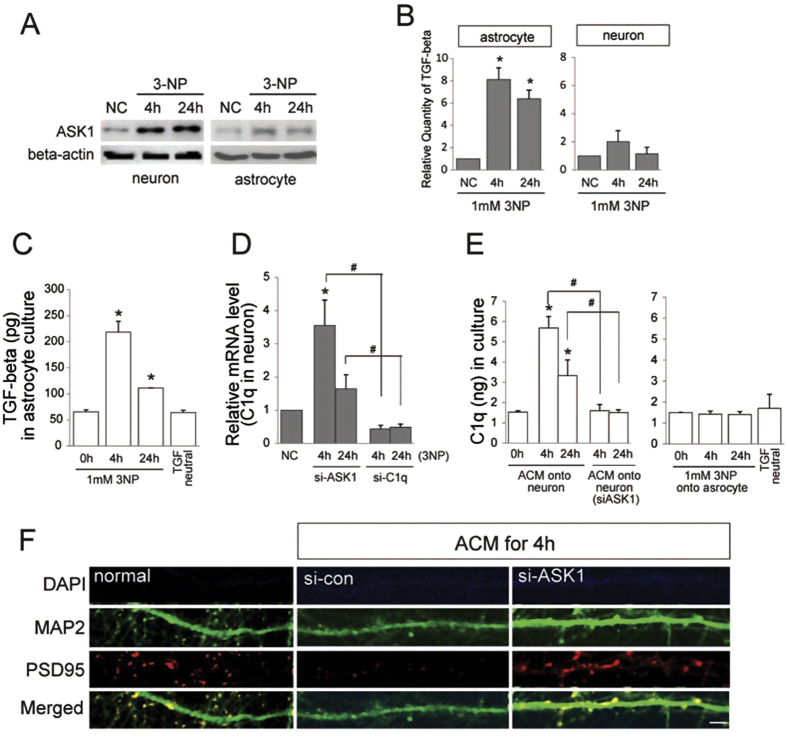
TGF-beta is differentially expressed in brain subregions, and astrocyte-derived TGF-β1 by 3-NP involves ASK1. (**A**) ASK1 protein levels were evaluated by Western blot in astrocytes (C8D1A) and primary neurons exposed to 1 mM 3-NP for 4 h and 24 h. (**B**) The transcriptional profile of TGF-beta was measured by qPCR in the astrocytes and primary neurons. (**C**) TGF-β1 secretion levels were assayed in the astrocyte by ELISA in a concentrated culture supernatant. The downregulation of ASK1 did not lower the levels of TGF-beta transcripts. (**D**) Neuronal C1q transcripts were highly expressed following 3-NP exposure, and the levels of C1q were reduced by ASK1downregulation. (**E**) Astrocyte-conditioned media (ACM) containing TGF-β1 can affect neuron cytotoxicity. When the neuron was replaced by prepared ACM and then incubated for another 4 h, neuronal C1q transcripts were highly expressed. However, ACM neutralized with the TGF-β1 antibody did not trigger an increase in C1q transcriptional levels. (**F**) When neuronal media were replaced by ACM, changes in the neuronal dendrites were demonstrated by immunocytochemistry with anti-PSD95. Scale bar: 5 μm.

To further evaluate the effect of TGF-β1 on neurons, astrocytes and primary neurons were exposed to chronic and mild 3-NP for intoxication (1 mM). The ASK1 protein was increased both in astrocytes and neurons following 4 h and 24 h exposures to 3-NP (Fig. 5A). TGF-β1 mRNA expression was evaluated in astrocytes and primary neurons using qPCR (Fig. 5B). Astrocytes and neurons exposed to 3-NP exhibited an increase in the expression of TGF-β1. ELISA revealed that TGF-β1 secretion increased in response to 3-NP with higher levels observed during the 4 h than the 24 h exposure (Fig. 5C). To evaluate whether TGF-β1 in ACM can induce C1q form neuron, astrocyte-conditioned media (ACM) was prepared after for 4 h and 24 h exposure to 3-NP. The neuron was replaced by prepared ACM and then incubated for another 4 h. The qPCR results revealed that neuronal C1q transcripts were highly expressed under ACM. However, when ASK1 was downregulated in the neuron, neuronal C1q transcripts were decreased even under ACM (Fig. 5D). Under ACM for 4 h, the C1q of the neuron was induced whereas neuronal C1q did not sufficiently trigger under ACM neutralizing TGF-β1 (Fig. 5E). Furthermore, when ASK1 was downregulated in the neuron, neuronal C1q transcripts decreased even under the ACM containing TGF-β1. When the neurons were replaced by ACM containing TGF-β1 for 4 h, neuronal cell death was not detected, but some detrimental changes of the neuronal dendrites were observed by anti-PSD95 immunostaining (Fig. 5F).

### The distribution of secreted C1q and TGF-β1 are differently regulated to the region

**Figure 6:**
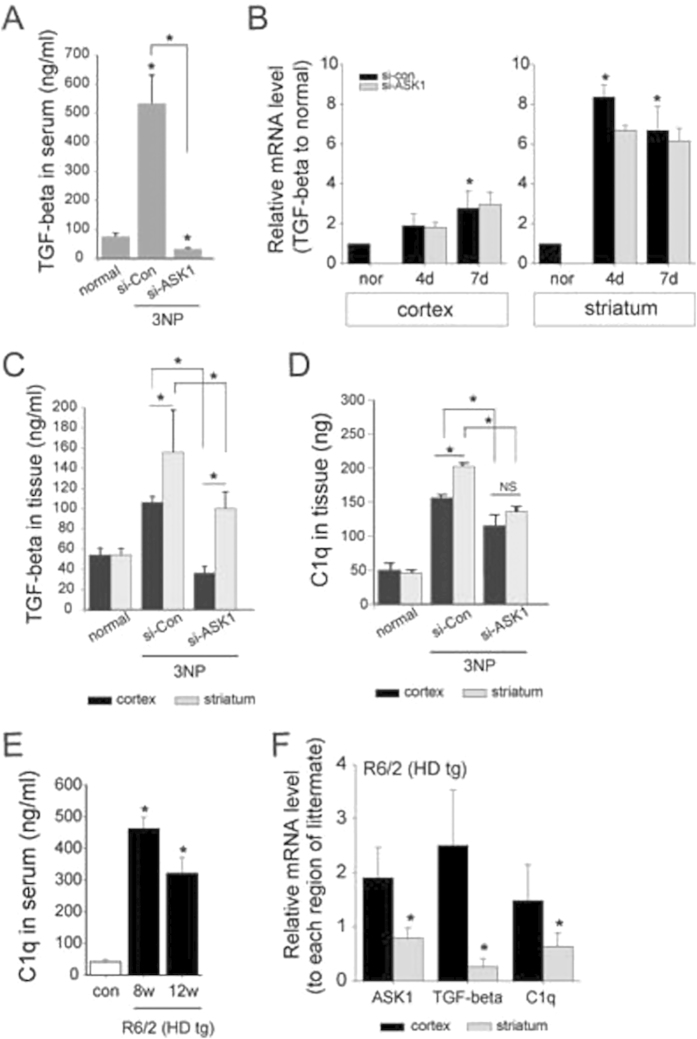
The levels of C1q and TGF-β1 change in response to 3-NP infusion. (**A**) The secretion level of TGF-β1 was also assessed by ELISA in 3-NP-infused mouse blood serum. TGF-beta was secreted at higher levels on day four than on day seven. When ASK1 was downregulated, TGF-β1 secretion was significantly decreased. (**B**) The transcripts of TGF-beta evaluated by qPCR shows TGF-beta of striatum to increase more than cortex. ASK1 downregulating did not affect to reduce the TGF-beta transcripts. (**C**) The ELISA results from 3-NP-infused for four days mouse brain tissue revealed that TGF-β1 increased on day four and remained elevated at a reduced level on day seven. The levels of TGF-β1 were higher in the striatum than in the cortex. The downregulation of ASK1 reduced TGF-beta levels in both the striatum and cortex. (**D**) The ELISA results from 3-NP-infused mouse brain tissue revealed that C1q was increased on day four and remained elevated at a reduced level on day seven. The C1q level was evaluated by ELISA separately in the striatum and cortex. ELISA also was used to assess C1q in the group of mice that received striatal C1q treatment as well as the group of 3-NP-infused mice in which ASK1 was downregulated. (**E**) In R6/2 HD tg mice, the secreting C1q level shows to rise in 8 weeks old and 12 weeks old HD tg. (**F**) The relative levels of transcripts of ASK1, TGF-beta, and C1q were measured by qPCR in R6/2 mice (10-week-old). The three kinds of transcript were differentially regulated in striatum and cortex. *p < 0.05.

We assayed TGF-β1 secretion level that is the potential factor of astrocyte-derived molecules in neuronal loss (Fig. 2A). The secretion levels of TGF-β1 demonstrated a similar temporal pattern to those of C1q, with a higher level on four days after 3-NP infusion than on day seven. The downregulation of ASK1 reduced the levels of TGF-β1 secretion (Fig. 6A). The qPCR results to evaluate the regional and temporal expression pattern indicated that the mRNA level expressed more in the striatum than the cortex. TGF-β1 transcript highly increased from day four in striatum while cortical TGF-β1 transcript was considerably increased only at day 7. Moreover it showed that down-regulating ASK1 did not lower TGF-β1 transcript at least (Fig. 6B). Nevertheless, down-regulating ASK1 showed neuroprotective effect in striatal neuron. In addition, the downregulation of ASK1 reduced the levels of TGF-β1 secretion more in the striatum than the cortex in 3-NP infused mice for four days (Fig. 6C). Correspondingly, the downregulation of ASK1 hampered C1q secretion in the whole blood serum (Fig. 3D). In each cortical and striatal tissue lysates, C1q was also assayed with ELISA indicating the pattern corresponding to the TGF-β1 (Fig. 6D). Overall, these results demonstrate that the downregulation of ASK1 reduces both secretion level of TGF-β1 in astrocyte and C1q in neuron. Moreover, these changes were proceeding to definitely form lesion. It could be a good prediction marker if these molecular changes are detected in the neurodegenerative disease HD which has character as a selective striatal lesion. Confirmatively, the result of ELISA presented the secreted C1q to raise at 8-week-age the early onset stage, and then decline the level at 12-week-age the disease developed stage (Fig. 6E). After confirming the Clq level according to disease developing stage, we evaluated what the mRNA of ASK1, TGF-β1, and C1q is expressed between cortex and striatum of HD tg mice at the age of 10-week. All three kinds of molecules are more expressed in the cortex than the striatum (Fig. 6F).

## Discussion

Our previous results suggested that changes in the levels of ASK1 may underlie the differential vulnerability of striatal neurons to 3-NP infusion^24^ or HD^25^. The 3-NP infusion mouse model was used in this study because a treatment could be concomitantly added at the beginning of pathogenesis. The results of the present study investigated whether innate immune factor, regarding as an important factor in neurodegenerative diseases^7^, participates in apoptotic cell clearance and weak synapse elimination in the 3-NP induced selective striatal lesion. Our findings are mainly that ASK1 is an initial participant in the pathogenesis of striatal neurodegeneration by 3-NP infusion and a crucial applicant in the formation of selective striatal lesions. The results of the present study is (1) chronic exposure to 3-NP secreted C1q differentially in each cortex and striatum, which was modulated by differentially expressed TGF-beta from astrocytes in each cortex and striatum; and (2) consequently selective striatal degeneration was occurred by ASK1 regulating C1q levels via astrocyte-derived TGF-β1during the early stages of lesion formation by chronic 3-NP infusion.

Although the systemic administration of 3-NP causes selective degeneration of neurons in the lateral striatum, few studies have infused 3-NP into mice using a subcutaneous osmotic pump, which differs from previous studies on rats^2^,^3^,^26^. The chronic systemic administration of 3-NP in rats and non-human primates homogeneously inhibit the succinate dehydrogenase (SDH) within the brain not solely the striatum^3^,^27^. And it is presented in this present animal model (Fig. 1). The evident striatal degeneration is occurred, which is associated with behavioral abnormalities that are reminiscent of HD, neurodegenerative disease^28^,^29^,^30^. In the current study, systemic 3-NP infusion was followed by increase in ASK1 protein and its active form levels in cortical region in spite of the lesion forming in striatal region (Fig. 1). The silencing of the ASK1 gene ameliorated striatal atrophy in the ipsilateral side infused with siRNA, as observed by MRI; eventually, motor function also was improved (Fig. 3).

During chronic and mild insults, the sustained exposure of neurons to immune mediators can bring neuronal dysfunction and contribute to cell death. Our preliminary data indicated that the transcriptional level of C1q is differentially changed by some brain injuries. When the brain is mildly and chronically exposed to 3-NP, C1q levels was upregulated in the serum. Moreover, the level was higher in the early stage than the late stage of striatal degeneration (Fig. 2). C1q, a complement factor in the classical innate immune pathway, and the dysregulation of complement proteins and cytokines has been demonstrated in many CNS disorders, including epilepsy^31^,^32^ and neurodegenerative diseases^33^,^34^,^35^. It has been proposed that C1q binds to weaker synapses and tags them for elimination^16^. Corresponding to the striatal loss occurring spot, astrocytes were strongly reactivated in the lateral striatum. It brought us to explore the astrocyte and the relationship between astrocyte and neuron. Although the signals controlling C1q expression and function remain elusive, it is reported that an unidentified secreting signals from reactive astrocytes upregulate expression of C1q in neurons. The very recent study reported that retinal TGF-beta as a key regulator of neuronal C1q expression and synaptic pruning in the developing visual system^36^. C1q is rapidly upregulated by astrocytic TGF-β1, and TGF-β1 is essential to that upregulation. The control of the C1q levels by TGF-β1 is decisive and specific in retinal development and eye-segregation^37^. We put the hypothesis on as (1) ASK1 is involved in striatal astrocyte reactivation; (2) reactive astrocyte secretes molecules detrimental to neuron; and (3) Striatal neurons are more susceptible to these factors. It is also consistent to our hypothesis that TGF-beta secretion level increased to similar pattern of C1q level (Fig. 2). Our results from C1qR immune-staining in the mouse brain revealed the local distribution of C1q (Fig. 4). The downregulation of ASK1 reduced C1qR-positive cells in the striatum compared with an increase in the C1q-treated brain. The 3-NP profile appears to be substantially attributable to astrocytosis, which is a common component of HD but is not HD-specific or necessarily a significant feature of early HD^38^. Our results also demonstrated that astrocytes were highly reactive in the lateral striatum for seven days following the infusion with 3-NP (Fig. 2). We proposed that ASK1 may differentially regulate C1q secretion levels via active astrocyte-derived TGF-β1 in the cortex and striatum, and is consequently involved in the axonal degeneration of corticostriatal projection neurons and subsequent striatal dendritic withering.

We supposed that striatal lesion formation results from the non-autonomous events as described above. In a previous study that examined TGF-beta signaling, serine-threonine kinase receptor-associated protein (STRAP), which is identified as an interacting partner of ASK1, was proposed to interact with TGF-β1 receptors and block TGF-β1 signaling^39^. ASK1 phosphorylated STRAP, and the inhibition of ASK1 suppressed the hydroxyl peroxide-induced apoptosis mediating STRAP by directly interacting between ASK1 and STRAP. The regulatory role of ASK1 in C1q levels may be consistent with this report that ASK1 is working as a negative regulator of the STRAP activation of TGF-β1 signaling. GFAP-positive astrocytes are distributed in the neonatal brain with a similar regional heterogeneity. Although the GFAP-positive astrocytes exhibited a different pattern during the developmental period and did not disappear at the postnatal stage, the regional allocation of reactive astrocytes may be induced by injury^37^. In the current study, TGF-beta exhibited a similar pattern to C1q on the days following 3-NP infusion. Although the downregulation of ASK1 did not reduce astrocytic TGF-β1 transcription levels (data not shown), the secretion level of TGF-β1 was effectively regulated by ASK1 (Fig. 6). Although it is needed the further study, the qPCR with brain tissue and ELISA with whole blood serum were assessed to differentially expressing pattern in cortex and striatum each (Fig. 6). It is implicated that the change of C1q level is applying to HD moreover could be representing the disease progress.

During chronic infusion of 3-NP, the sustained exposure can weaken neuron synapses, leading to neuronal dysfunction and finally striatal neuronal cell death. In the striatal neurodegeneration derived by 3-NP infusion, the C1q level is increased at a relatively early stage of degeneration before the lesion or abnormal dysfunction is observed. Therefore, the results of the present study suggest that the C1q level in the blood may be a surrogate prediction marker representing disease progression, such as in HD. It can be used for prognostic purpose for the development of behavioral symptoms or cognitive impairment.

## Materials and Methods

### Primary neuronal cell culture

Primary neurons were cultured with mouse pups and culture procedures are based on the widely used, non-feeder layer–based protocol. Embryonic day 14 pups were removed from the pregnant mother mouse. Cortex and striatum was dissected in Hank’s buffered salt solution (CMF-HBSS) and tissues were digested. The supernatant was removed, rinsed with three successive washes of HBSS and resuspended in neurobasal media (NB, Life Technologies, USA) contained 0.2 mM GlutaMAX (Life Technologies, USA) and 2.0% B-27 Supplement (Invitrogen, USA). Cells were subsequently plated on multi-well plates or dish pre-coated with 1 mg/mL poly-L-lysine (Sigma, Milwaukee, WI, USA) at a density of 5 × 10^4^ cells per mL. Neurons were subsequently incubated at 37 °C with 5% CO_2_ and remained in culture for 15 days *in vitro* (DIV). Every 3–4 days, the neurons were fed by replacing one half of the fresh NB media.

### Animal model and lesion analysis

In this study, 10-week R6/2 HD tg (The Jackson Laboratory, Bar Harbor, ME, U.S.A.), littermate wt, and male C57BL/6J mice (SLC, Inc., Shizuoka, Japan) were used. All procedures were performed in accordance with guidelines for the care and use of laboratory animals at Yonsei University, as approved by the Association for Assessment and Accreditation of Laboratory Animal Care (AAALAC). Mice were anesthetized with 2.0% isoflorane under 30% oxygen and 70% nitrous oxide using a vaporizer (VMC Anesthesia Machine, MDS matrix; Orchard Park, NY, USA). 3-NP (Sigma, USA) was dissolved in saline to a concentration of 0.5 mg per μl (pH 7.4). The prepared solution of 3-NP was delivered by sustained infusion (180 ~ 190 mg/Kg/day) using osmotic minipumps at an infusion rate of 0.5 μl/h for seven days (1007D; Alzet, Cupertino, CA, USA). C1q (Quidel, San Diego, CA, USA) (100 ug/ml, 2 ul) was injected into striatum (anterior, 0.7 mm; lateral, 1.2 mm; depth, −3.3 mm) for 5 min and withdrew the needle after retention for 7 min. The degenerated brain region was validated with cresyl violet staining, and magnetic resonance image (MRI) analysis. For MRI analysis, we performed MRI scans of mice on a horizontal bore (400 mm) 4.7 Teslar MR scanner (Brucker Biospin, Billerica, MA, USA) after mice were anesthetized with isoflorane. Images were obtained using a T2-weighted fast spin echo sequence. Diffusion images were also acquired. Temperature was maintained and respiration was monitored throughout the entire scan. Mice recovered quickly following the scan with a 100% survival rate. The striatal atrophy was measured as an area of the lateral ventricle.

### Behavior and motor function test

The motor functions of the mice were tested with the hindlimb clasping function. Mice were suspended by their tail and checked whether their hindlimb were clasped or not. The accelerating rotarod was performed on a rotarod apparatus (Ugo Basile North America Inc., Schwenksville, PA, USA) with speeds that varied from 4 to 40 r.p.m. for a maximum of 5 min. Each mouse was pre-trained prior to the implantation of the osmotic pump filled with 3-NP or saline. The training session was followed by a 30 min rest period, after which mice were placed back on the rotarod for five trials of five minutes maximum at accelerating speeds.

### qPCR

Total RNA was isolated from mice striatum and cortex. Isolated RNA was purified and its quality determined by NanoDrop. Total RNA (200 ng) was used in real-time quantitative PCR (qPCR) using a StepOne Plus (Applied Biosystems, USA). Reactions were performed in a 20 μl volume with 2 μl cDNA, 0.5 μM primers and reagents included in the DNA Master SYBR Green I mix (Roche Diagnostics, Germany). The reaction underwent pre-incubation step at 95 °C for 5 min, and was amplified by 45 cycles of denaturing at 95 °C for 15 sec and annealing and extending at 60 °C for 30 sec. Detection of the fluorescent products was carried out at the end of the extension period. GAPDH was amplified as an internal control. To confirm amplification specificity, the PCR was performed in no-transcript-control (NTC) and the PCR products from each primer pair were subjected to melting curve. Data analyzed by the comparative Ct method. Each gene was analyzed in three biological replicates and three technical replicates.

### Immunocytochemistry/immunohistochemistry and confocal microscopy

To determine the pattern of localization of each molecule, we performed immunofluorescent staining. After sacrificing the mice, the brains were removed, postfixed overnight in 3.7% formaldehyde, and stored in 30% sucrose. For cultured neuron fixation, neurons on a coverslip fixed with 4% sucrose in 2% PFA for 10 min. After fixation, the brains were cut into coronal sections of 20 μm thickness on a cryostat section and processed for immunohistochemistry. Fixed sections were incubated with blocking solution, and incubated with a primary antibody at a dilution of 1:100. After washing, the sections were incubated with fluorescence-conjugated antibody (1:200; Jackson ImmunoResearch, West Grove, PA, USA). For counter-staining, the sections were incubated with DAPI or Hoechst 333342 (Molecular Probe, USA). After washing, stained tissue samples were mounted using Vectashield (Vector Lab, Burlingame, CA, USA). These sections were then observed under a confocal laser scanning microscope (Carl Zeiss, Thornwood, NY, USA).

### Cell death assay

According to manufacturer instructions, apoptosis-related DNA fragmentation assay was evaluated using a cytoplasmic histone-associated DNA fragment kit (Roche Diagnostics, USA). We performed assay following the manufacturer’s protocol.

### Gene silencing

The ASK1 gene was silenced using siRNA against ASK1 (sense, GCUCGUAAUUUAUACACUGtt; antisense, CAGUGUAUAAAUUACGAGCtt; conc: 5 μM; Ambion, Austin, TX, USA). The silencing efficiency was confirmed in PC12 culture cells. After confirmation *in vitro*, the transfecting reagent, SiPORT *Neo*FX (Ambion), and ASK1-siRNA or nonfunctional mutant RNA (control siRNA, 5′-AAG AGA AAA AGC GAA GAG CCA-3′; Ambion) were combined, mixed gently, and allowed to form siRNA liposomes for an additional 10 min at room temperature. A micro-osmotic pump (Alzet, USA), containing 100 μl of the transfection reagent only or ASK1-siRNA was then placed subcutaneously on the backs of the mice and a brain infusion cannula connected to a pump was positioned at the intra-striatum. The designated solution was infused for seven days at a rate of 0.5 μL/h during 3-NP infusion. The mice were killed seven days after surgery and their brains were processed. In case of primary neuron, siRNA was tansfected at 12 DIV and collected at 15 DIV after incubating for 72 hours.

### Astrocyte conditioned medium

To test the effect of astrocyte derived unknown factor on neuron, we used astrocyte (C8-D1A) which was purchased from American Type Culture Collection (ATCC, Manassas, VA, USA). Astrocytes were cultured in Dulbecco’s Modified Eagle’s Medium high glucose (DMEM, Invitrogen, Carlsbad, CA, USA) supplemented with 10% fetal bovine serum (FBS, Invitrogen, USA) and 1% penicillin-streptomycin solution (Thermo Scientific, Waltham, MA, USA). The cultures were maintained at 37 °C in a humidified CO_2_ incubator. For obtaining astrocyte conditioned medium (ACM), confluent astrocyte cell cultures were washed and replaced with serum-free medium DMEM/F12 (Thermo Scientific, USA) without FBS (Invitrogen, USA). Astrocytes in serum-free media were exposed to 1 mM 3-NP for 4 h and 24 h each. After then, media were collected and removed cell debris by centrifuging at 1,000 *g* × 10 min. To evaluate the effect of ACM on neurons, primary neurons were cultured in Neurobasal media (Invitrogen, USA). At 15 DIV, neurons were replaced with ACM exposed to 3-NP and incubated additionally 4 h. Effects of ACM on neuron were assessed by immunocytochemistry of PSD95.

To further evaluate whether TGF-β1 of ACM mainly affected on neuron and synaptic dysfunction, ACM was incubate with 1 μg/mL TGF-β1 antibody (Abcam, Cambridge, MA, USA) for 30 min at room temperature to deplete TGF-β1 from ACM by neutralizing the TGF-β1. ACM neutralizing TGF-β1 was also treated on neuron for 4 h and neuronal changes were observed. According to the manufacturer’s information, TGF-β1 is neutralized 50% of the bioactivity when 0.25 μg/mL TGF-β1 antibody was treated in HT2 cell line.

### ELISA

The samples for ELISA were prepared in animal blood serum and culture media. Prepared samples were performed according to the manufacturer’s provided protocol. Standards were diluted with each assay buffer provided. Each assay was followed with each procedure. ELISA kits were obtained for total TGF-β1 (BioLegend, San Diego, CA, USA) and mouse C1q (Hycult Biotech, Uden, Netherlands).

### Statistical analysis

Data are expressed as mean ± SD. The significance of differences among multiple groups was evaluated by ANOVA followed by Bonferroni’s *post hoc* protected least-significant difference test while two groups were compared using unpaired *t* tests (SigmaStat, Systat Software Inc, San Jose, CA, USA).

## Additional Information

**How to cite this article**: Cho, K. J. *et al.* Apoptosis signal-regulating kinase 1 mediates striatal degeneration via the regulation of C1q. *Sci. Rep.* **6**, 18840; doi: 10.1038/srep18840 (2016).
